# Assessing Risks from Cyclones for Human Lives and Livelihoods in the Coastal Region of Bangladesh

**DOI:** 10.3390/ijerph14080831

**Published:** 2017-07-25

**Authors:** Mohammad Abdul Quader, Amanat Ullah Khan, Matthieu Kervyn

**Affiliations:** 1 Department of Geography, Earth System Science, Vrije Universisteit Brussel, 1050-Brussels, Belgium; makervyn@vub.ac.be; 2 Department of Geography and Environment, Jagannath University, Dhaka-1100, Bangladesh; 3 Department of Geography and Environment, Dhaka University, Dhaka-1000, Bangladesh; amanat.ullah@gmail.com

**Keywords:** exposure, hazard, vulnerability, capacity, risk map, casualty, cyclone, cyclone shelter, PCA, Bangladesh

## Abstract

As a disaster prone country, Bangladesh is regularly hit by natural hazards, including devastating cyclones, such as in 1970, 1991 and 2007. Although the number of cyclones’ fatalities reduced from 0.3 million in 1970 to a few thousand or fewer in recent events, loss of lives and impact on livelihoods remains a concern. It depends on the meteorological characteristics of cyclone and the general vulnerability and capacity of the exposed population. In that perspective, a spatially explicit risk assessment is an essential step towards targeted disaster risk reduction. This study aims at analyzing the spatial variation of the different factors contributing to the risk for coastal communities at regional scale, including the distribution of the hazards, exposure, vulnerability and capacity. An exploratory factor analysis method is used to map vulnerability contrasts between local administrative units. Indexing and ranking using geospatial techniques are used to produce maps of exposure, hazard, vulnerability, capacities and risk. Results show that vulnerable populations and exposed areas are distributed along the land sea boundary, islands and major inland rivers. The hazard, assessed from the density of historical cyclone paths, is highest in the southwestern part of the coast. Whereas cyclones shelters are shown to properly serve the most vulnerable populations as priority evacuation centers, the overall pattern of capacity accounting for building quality and road network shows a more complex pattern. Resultant risk maps also provide a reasonable basis from which to take further structural measures to minimize loss of lives in the upcoming cyclones.

## 1. Introduction

Cyclones are one of the most destructive natural hazards along highly populated coastlines all over the world. This is especially true for low-lying delta environments such as Bangladesh. A cyclone hits the coast of Bangladesh almost every year. Among these, at least one severe cyclone every three years causes significant damage along part of the coast [1]. The majority of the coastal inhabitants in Bangladesh are poor, landless and depend on natural resources. Loss of lives and livelihoods; damage to agriculture, infrastructure and settlements; and disruption of communication networks are the major impacts of cyclones in Bangladesh [2,3].

Human deaths and injuries due to cyclones, often associated with storm surges, are one of the major concerns to the national, regional and local policy makers in Bangladesh. Although the number of casualties caused by recent major cyclones (i.e., in 2007, 2009, and 2016) decreased compared to those in 1970 and 1991, the probability of human loss due to future cyclones remains significant [4]. Understanding the spatial distribution of the vulnerability, capacity, exposure and resultant risk of coastal communities is important to reduce future human casualties.

Risk is the probability that an extreme natural hazard cause adverse impacts on vulnerable elements exposed to it, including loss of lives, properties or other economic assets [5,6,7]. It has been conceptualized as the combination of a hazard, exposed elements and their specific vulnerability, sometimes moderated by the capacity of the exposed elements to face the hazards.

Vulnerability, usually considered as two components namely physical and social vulnerability, refers to the susceptibility of people, sector of society or economy to death, injury or damage by natural hazard [8,9,10,11,12,13,14,15]. Assessment of vulnerability has been proposed by developing indices based on different spatio-temporal scales, hazard types and their impacts [16,17,18,19,20,21,22]. Capacity refers to the ability of a community or system to face, cope and recover from the adverse effect of natural hazards [3,23,24]. It involves factors such as technology, knowledge, institutional strengths, access to information, social networking, and physical assets [23,25,26,27]. In Bangladesh, cyclone shelters have been proposed as a key risk reduction measures [28,29]. Although there are other aspects controlling the capacity of communities to resist a disaster, such as their political power and social networks, it is difficult to assess them quantitatively. Therefore, in this study, we considered only physical capacity components such as cyclone shelter, housing structure and communication network.

Exposure is defined as the degree to which a place is exposed to natural hazards [30]. Potential indicators of it are characteristics of the habitat of people, density of population, livelihood and environment [23,31,32,33,34]. Hazard is the probability of occurrence of a physical event that have potential to cause damage or loss to human lives and properties [35,36]. Intensity based on wind speed at the time of landfall and return period are often used as key characteristics for cyclone hazard assessment [37,38].

This paper presents a first effort to assess regional risk to cyclone casualties and damage at the scale of the lowermost administrative level of Bangladesh. The main objective of the research is to identify the spatial distribution of cyclone risk for the communities living in coastal areas of Bangladesh. The specific objectives include the mapping of the spatial distribution of the exposed population, the main vulnerability, hazard and capacity factors and their integration into a risk map.

Using available socio-economic statistics for unions’ administrative level (4th order administrative level of Bangladesh that comprises several villages), historical cyclone paths and geospatial analysis, we propose regional maps of cyclone hazards as well as exposure, vulnerability and capacity indices of the coastal population. These indices are derived using principle component analysis (PCA), weighted indexing and ranking with the help of geospatial techniques. Vulnerability with its contributing dimensions; and capacity, exposure and hazard with their contributing components are mapped to understand how they are spatially distributed. As vulnerability, capacity, exposure and hazard contribute to the risk of natural hazard [7,32,36,39], they are aggregated in different arithmetic combinations to produce four types of risk maps of the study area. The main challenge of this spatial risk assessment, similar to other parts of the world [40,41,42,43,44,45], is the validation of the vulnerability and risk maps produced. Despite the data scarcity of past cyclone fatalities at the scale we are working, we attempt to compare the spatial patterns of our vulnerability and risk maps with a damage map for the 2007 cyclone.

## 2. The Coast of Bangladesh and Historical Cyclones

**Figure 1:**
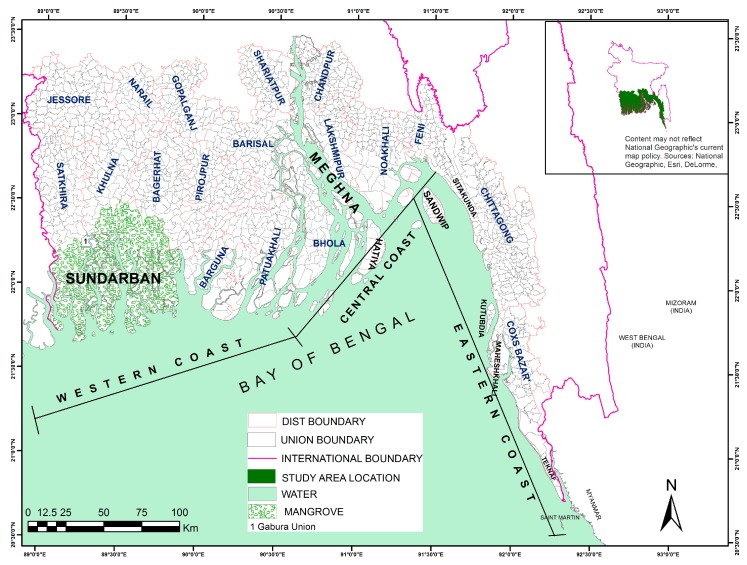
Map of the Study area highlighting the main geographic features discussed in the text. Inset highlights the location of the coastal area within Bangladesh. Boundaries of all unions are displayed, as well as the names of the districts (2nd order administrative level), the major river, and major islands are displayed. Three coastal zones are drawn after MCSP, 1993.

Bangladesh is a land of natural hazards. Cyclones form in the Bay of Bengal (Figure 1) and regularly hit the coast of Bangladesh. The coast of Bangladesh shows three distinct zones i.e., western coast with mangrove forest, central coast with numerous geomorphological active offshore islands and eastern zones with higher elevation (Figure 1). The coastal area is characterized by low topography except for the steeper eastern part and is formed from alluvial deposition of three main rivers, namely the Ganges, Brahmaputra and Meghna [46,47]. The Integrated Coastal Zone Management Plan of Bangladesh (ICZMP) identifies 19 districts (second order administrative unit of Bangladesh) as coastal area based on tidal fluctuations, soil, surface and underground water salinity, and cyclone-affected areas [48]. The coastal area of Bangladesh covers an area of 40 × 10^3^ km^2^, with 710 km of coastline [49], many islands and river channels. It is characterized by a high population density of 723 per square kilometer.

**Figure 2:**
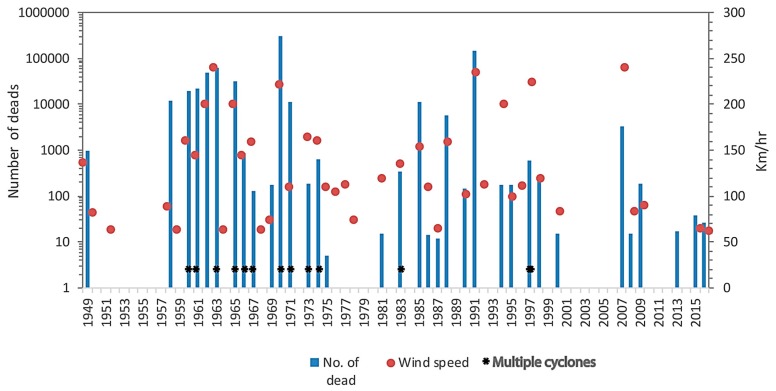
Historical cyclones and losses of lives (1948–2016). Wind speed is reported at the time of land fall of the cyclone. The data have been compiled from different sources such as Bangladesh Meteorological Department (BMD), published and unpublished papers, unclassified reports of the government and daily newspaper. In the case of conflicting information between sources, we relied on Government statistics. There were no data of human casualties or wind speed for several of the listed cyclones. Cyclone data that have missing information of both human casualties and wind speed have been excluded from the graph.

Since 1797, 70 cyclones of different intensities caused the death of ca. 1.5 million people in Bangladesh according to the compilation of different sources [50,51,52]. Among them, 18 cyclones were severe to most severe in nature based on the wind speed and associated loss of lives. Cyclones are often associated with tidal surges, which are the major cause of casualties (Figure 2).

Poverty, poor structure of housing, lack of adaptive capacity and nature-dependent livelihood of coastal people increase their vulnerability to cyclone and wind-driven storm surges [25,53]. The number of deaths from severe cyclones dropped from 300,000 in 1970 to 145,000 in 1991 (cyclone *Gorky*) and 3000 in 2007 (Cyclone *Sidr*). The number of casualties decreased as government took measures at policy level (i.e., enacting standing order on disaster) and implementation level (i.e., building polders and cyclone shelters, and cyclone preparedness program after the independence of the country in 1971). The number of casualties from cyclone depends on multiple conditions such as landfall time (day or night), synchronization with high tides, wind speed and proper dissemination of accurate warnings before landfall. Similar warning signals produce different cyclone impacts as assigned danger levels do not consider the variation among the characteristics of the people living along the coast, their exposure and ability to respond. Assessment of population exposure, socio-economic vulnerability, and capacity to find shelter are important to understand the differential impacts of the cyclones based on the spatial variations.

As the coastal unions are poorly connected to district and division headquarters, elected local representatives and government officials of the lowest administrative level (unions) should act promptly in the phases before, during, and after a disaster. Many risk reduction and management decisions are taken at union’s level, including the selection of beneficiaries of the social safety budget, as well as structural and non-structural risk reduction actions such as repairing polders or dissemination of warning messages [3]. Union disaster management committees are documented in standing order on disaster (SOD) to act locally to manage disaster in coastal area.

## 3. Materials and Methods 

### 3.1. Vulnerability Assessment

Building a vulnerability index requires the selection of meaningful variables as well as a method to aggregate these data into a single index. The choice of variables is more important than methods followed in index building [43,54].

**Figure 3:**
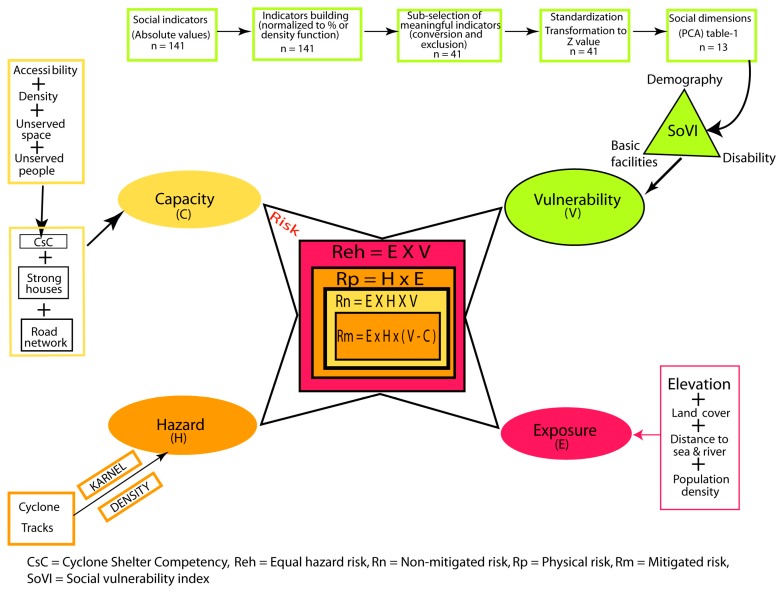
Flow diagram of methods of risk assessment. “*n*” means the number of variables used.

In this study, we applied data driven inductive approaches to assess the vulnerability of the population to cyclone casualties and damage at union level [19,44,54]. We used the latest available national census data of 2011 and poverty data of the World Food Programme to select the primary socio-economic indicators of the people living in the coastal unions. After careful examination of hundreds of available variables, 141 of them were selected for further processing (Figure 3). These variables provided information about factors considered in previous studies as important indicators to assess vulnerability such as household demography, education status, health and disability, income, profession, minority and ethnicity [55,56]. As the raw variables are in absolute values, they were transferred to percentage, average, ratio or density function. The 141 variables were again scrutinized and scaled to 41 indicators by transferring and combining some of them to meaningful widely-accepted social indicators (i.e., $\mathrm{dependency}\;\mathrm{ratio}=\frac{number\;of\;people\;aged\geq 65+number\;of\;people\;aged<15}{number\;of\;people\;aged\;15–64}$).

These 41 indicators have heterogeneous units. To make them comparable they were converted to standardized *z* values.

For further reducing the number of variables and analyzing multicollinearity, we applied a data reduction technique, Principle Component Analysis (PCA), using a varimax rotation option. The necessary preconditions for PCA application (selections of variables that have correlation coefficients < 0.8, Keiser–Meyer–Olkin (KMO) ≥ 0.5 were checked and met. The factors with eigen values greater than one were retained for calculating vulnerability indices. Each factor was attributed a directional sign based on the interpretation of the contribution of the dominant variables’ loading on the factor to increase or decrease vulnerability [9].

Finally, the social vulnerability index was calculated for each coastal union based on a weighted additive model using the retained PCA factors. The weight used in the additive model is calculated based on the relative variance explained by each factor (Table 1). The spatial pattern of the relative social vulnerability was mapped into five categories using quantile as cut points.

### 3.2. Capacity Assessment Against Cyclone

#### 3.2.1. Cyclone Shelters

Cyclone shelters have been argued to be the most important elements contributing to save human lives from the impact of cyclones [57]. The locations of cyclone shelters have been collected from the cyclone shelter information database (cyclone shelter database, 2016) and validated by collecting locations with a handheld GPS in six unions during field work in June 2015.

The ability of the cyclone shelters to save human lives has been assessed by four indicators: (1) the average distance of cyclone shelters to nearest roads; (2) the density of shelters; (3) the unserved space; and (4) unserved people by cyclone shelters.

The distance of each cyclone shelter to the nearest usable road was calculated using Euclidian distance. For each union, the average distance of the different shelters to the road was calculated. Unions having no shelters were attributed the largest distance values obtained for unions with at least one shelter. A negative value was attributed to this indicator, as the lower the average distance of shelter to the road, the more accessible they are to provide capacity against cyclone risks.

The density of cyclone shelter is calculated from the number of cyclone shelters per union divided by the area of respective unions. The service area of a cyclone shelter has been shown to be limited to 1.5 km radius due to accessibility constraints during cyclones [49]. Based on this, we calculated the fraction of the territory of each union which is located within 1.5 km of a shelter. This indicator was attributed a positive sign as a higher value indicates that a larger proportion of the union is within the reach of a shelter.

Each shelter is characterized by a certain capacity, i.e., the number of people that can find shelter in it. The population of each union and the standard capacity of cyclone shelter (i.e., considering two square feet area per person as standard space requirement) have been collected from national population census and cyclone shelter database, respectively. The proportion of the union’s population that can be accommodated in available shelters was then calculated as a ratio of total population.

This indicator was attributed a positive sign as a high number of people served by cyclone shelters indicate that higher capacity of the union to protect lives from cyclone threat.

The four shelters’ indicators were averaged using equal weight to calculate the “cyclone shelter competency index”.

#### 3.2.2. Housing Strength and Communication Suitability

Houses that are constructed with reinforced cement concrete (RCC) and bricks can withstand strong wind forces and storm surges. These houses are considered as strong houses in this study. Percent of strong houses per union has been collected from national population census data of 2011. This parameter was converted into *z*-value to give the “housing strength indicator”. This indicator contributed positively in increasing the capacity of a union to face the impact of a cyclone.

The mobility of population during cyclones is quite essential, to evacuate inland or reach cyclone shelters. Shape file of roads has been collected from WFP (The World Food Programme) dataset. The road density within each union has been calculated by dividing the total length of roads, considering all types of construction material (i.e., paved, brick-built, earthen), by the union area.

All of the above indicators were converted to *z*-value. A final capacity index was calculated by averaging the cyclone shelter competency index, the housing strength indicator and the road density.

### 3.3. Exposure Assessment

In the literature, a confusion exists between the concepts of exposure and vulnerability: some previously proposed coastal vulnerability Indices [22,58,59,60] exclusively consider geographic factors influencing the probability of an area to be affected by a hazard. We consider these geographic components as part of the exposure dimensions of risk. The land elevation, land cover, distance from the sea–land and river–land boundary, and population density are considered as exposure indicators in this study.

Land elevation is extracted from the SRTM DEM at 30 m resolution. Elevation is considered to contribute negatively to exposure, assuming higher elevation induces lower exposure to tidal surge and flooding [22,58,59].

We used a global land cover dataset prepared from Landsat satellite images at 30 m resolution [61]. We updated this land cover map with the vector maps of rivers and built up areas at a scale of 1:50,000 prepared by the Local Government Engineering Department of Bangladesh. We assigned exposure ranking to each of the eight land cover categories based on available literature (Table S1), considering the economic importance of each land cover and its sensitivity to cyclone’s impact.

Distance of each point of the study area to the closest river and coastline were calculated separately and added together later. Distance to coast and river were assigned a negative sign—meaning that they contribute negatively to exposure—assuming the places closest to water are the most exposed to flooding and tidal surges.

We converted all the four indicators to z factor. As these factors are poorly correlated with each other and the relative importance of each of them is ill-defined, we aggregated these indicators using an arithmetic average, with equal weight to each indicator, to construct the exposure index. The unions were mapped using exposure indices and ranked into five categories based on quantiles as cut points.

### 3.4. Hazard Assessment

The cyclone tracks of historical cyclones in Bangladesh have been collected from NOAA global cyclone dataset and updated with the cyclone tracks provided by Bangladesh meteorological department. The kernel method of linear features, with a radius of approximately 2.5 km i.e., equivalent to 1/30 of the north–south dimension of the study area, was used to derive a probability density function of all the cyclone tracks [62]. This simple approach does not account for the actual wind speed and size of each cyclone cell, nor for the variation in maximum wind speed along the cyclone track, due to lack of detailed data on these parameters.

### 3.5. Cyclone Risk and Impact

Several methods can be used to assess the risk to natural hazards as different combinations of Vulnerability, Capacity, Exposure and Hazard. We applied four of these approaches. As the variables, indicators, indices and dimensions, used in assessment of risk to natural hazards, vary based on availability of the data in different parts of the world, there is always uncertainty of the produced risk map. To minimize those, we used several possible combinations of risk components to prepare risk maps of different alternative scenarios. Although individual risk indices exist as theoretical definitions in the literature [63,64] and are used separately in other parts of the world for specific case studies [7,9,41,42], the application of four risk indices in the same case study is indeed an innovative component of our study. We consider different meaningful arithmetic combinations of these components to map the spatial pattern of risk in the coastal unions (Figure 3). First, we produced a “Equal hazard risk index” taking into consideration the vulnerability of the people and exposure of the place where they live but assuming equal spatial distribution of hazard as the track of future cyclone is unknown (Equation (1)). The second “Non-mitigated risk index” considered the product of hazard, vulnerability and exposure, assuming the historical spatial distribution of hazard to be valid for the year of study (Equation (2)). The third “Physical risk index” considered only the hazard and exposure components, neglecting the socio-demographic characteristics of the population, and therefore focusing on the spatial distribution of physical impacts (Equation (3)). The final “Mitigated risk index” considered capacity of the people in addition to the three other components (Equation (4)). We model the mitigated cyclone risk by subtracting union’s capacity from its vulnerability, before multiplying with exposure and hazards. Risk equations are normally a multiplication (i.e., Risk = Hazard × Vulnerability or Risk = Hazard × Exposure × Vulnerability/Capacity), so that if one component of equation is zero the risk become zero [64]. We did not divide vulnerability by capacity to avoid producing undefined value in the case of any union without capacity (i.e., capacity index = 0).

Equal Hazard Risk = Vulnerability × Exposure(1)

Non-mitigated Risk = Vulnerability × Exposure × hazard(2)

Physical risk = Exposure × hazard(3)

Mitigated Risk = Exposure × Hazard × (Vulnerability − Capacity)(4)

The damage data of cyclone *Sidr* that hit Bangladesh in 2007 have been collected from the needs assessment report produced by the Disaster Management and Relief Department of Bangladesh [65]. A cyclone impact map was prepared based on the converted *z*-values of human death, injury, livestock and property damage data at a district scale to use as a proxy for cyclone impact. As the scale of these damage statistics is rougher and as it concerns a single cyclone event, only semi-quantitative comparison with the produced risk map is conducted.

## 4. Results

### 4.1. Factors of Vulnerability

Using the PCA and the 41 initial statistical indicators, thirteen vulnerability factors have been extracted to differentiate the coastal unions of Bangladesh by relative vulnerability (Table 1). The 13 retained factors all together explain ca. 79% of total variation among unions. These belong to three main characteristic groups of social vulnerability (dimensions), namely the socio-demographic and economic characteristics of the population, their access to basic facilities and the proportion of physically or ethnically marginalized people.

#### 4.1.1. Demographic, Education and Job Opportunities

**Figure 4:**
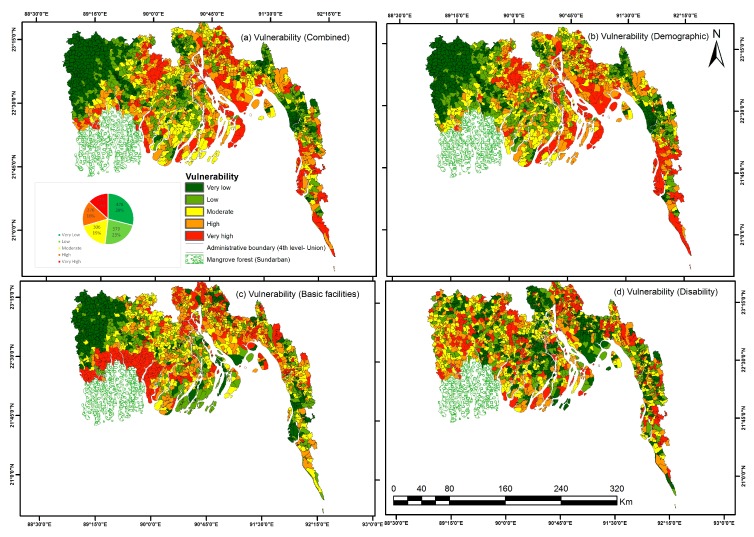
Vulnerability Map of Study Area. Vulnerability scores has been calculated based on 13 dimensions (Factors by PCA) from 41 social indicators. Maps of three major characteristic group of vulnerability are shown in: (**b**) Demographic; (**c**) Basic facilities; and (**d**) Disability. The overall vulnerability is shown in map (**a**) vulnerability (combined). Southwestern mangrove forest was kept out of analysis.

This vulnerability dimension regroups 6 components of the PCA explaining 51.6% of the total dataset variations. It is related to the demographic structure, education level and opportunities in different job sectors. Coastal unions along the south east coastline, all unions bordering main rivers in the central coastal region (i.e., Kutubdia Island, most of the unions of Hatiya Island, densely Chittagong port city) are highly vulnerable areas (Figure 4b). The population in the Western coastal part displays the lowest vulnerability in this dimension. The exception is Gabura Island union located just beside Sundarban. It is ranked as highly vulnerable on this dimension and was badly affected by cyclone *Aila* in 2009 [4]. High and very high vulnerable areas in the central coast are closely following the flow path of the lower Meghna River along the entire estuary length.

The first PCA component contributing to this vulnerability dimension is composed of indicators related to social security and citizen’s socio-economic opportunity that decrease vulnerability to natural hazards. People who have better jobs, better access to safe drinking water, better housing can recover earlier from cyclone impact than those that do not have such opportunities [66,67,68]. Higher education level increases the level of understanding to warning signals and the opportunity to get jobs that leads to lower vulnerability [53]. All these indicators load significantly on the first PC which summarizes 19.5% of the dataset variance among coastal unions. The second component explaining 9% of variance, relates to the demographic factors, including the size of households, the proportion of dependent people, and the family structure and marital status of female. There is general understanding that larger families, with large number of dependent people such as elders and children, are more vulnerable to cyclones as they need special assistance during evacuation [13,69].

Unemployment and education indicators are loading on the third component of this socio-demographic vulnerability dimension. Unemployed people have no formal income. Inaccessibility to formal education results in poor understanding of hazard forecasting and risk misperception. Both indicators increase vulnerability. Natural hazards are discussed at school from primary to higher secondary level in Bangladesh [3]. People out of schooling cannot get access to this information, making them vulnerable to natural hazards.

The sex ratio of the unions and percentage of male child aged over seven that are not enrolled in school are the indicators loading on the 5th component which is assumed to increase vulnerability. Higher sex ratio (females = 100) means gender imbalance that encourage early marriage of poor rural girls that eventually increase vulnerability [10].

The seventh component, accounting for 4.7% of the variance, concern industrial jobs. Industry workers are better paid compared to agriculture wages and other informal sectors [70,71] and can generally afford stronger houses, capable of providing higher protection during cyclones. As their job is less dependent on land resources, they are able to recover soon after a disaster because their source of income might not be affected.

Males and females of the unions who are looking for jobs are financially not capable to support their family in post cyclone recovery. These indicators are summarized in the 8th component that accounts for 4.5% of the total variance.

#### 4.1.2. Access to Basic Facilities

Access to basic facilities such as safe drinking water and hygienic toilets are important in controlling the health status of the population and propagation of diseases. There was a widespread crisis of drinking water and sanitation after cyclone *Aila* in 2009 in southwestern coastal districts. This second dimension of vulnerability is composed of the 6th (access to water) and 9th (access to toilets) components of the PCA, which account together for 8.8% of the total variance. Part of the southwestern coast area, surrounding the Sundarban mangrove reserve, is the most vulnerable in terms of limited access to these basic facilities (Figure 4c). Further north in the western coast, unions have much better access to these facilities. In other parts of the coast, there is no clear spatial pattern for this vulnerability dimension.

#### 4.1.3. Physically or Ethnic Marginalized People

This last dimension of vulnerability groups the PCA components highlighting specific marginalized groups associated with ethnic minorities or health disabilities. Composed of components 4, and 10 to 13 of the PCA, this vulnerability dimension explains 18% of the total variance among coastal unions. It includes components of different types of physical and mental disabilities that reduce capacities of affected people to understand and/or react properly during risk situations [72]. It also includes a component summarizing the proportion of religious and ethnic minorities (i.e., tribes and non-Muslims) that are often more vulnerable due to discriminations or limited political representations. Religious minorities and tribal ethnic groups indeed face institutional discrimination and are socially excluded in decision making process in Bangladesh [3]. Cyclone warning dissemination system is also not equal friendly to these socially excluded people as tribal people speak their own languages which is different than the official language of cyclone warning messages.

The vulnerability map for this disabilities and minorities dimension shows no specific spatial pattern (Figure 4d). Unions with high value on this dimension are scattered across the coastal areas and do not concentrate along the coastline.

### 4.2. Overall Vulnerability Assessment

The three vulnerability dimensions mentioned above were integrated in an overall vulnerability map, accounting for the proportion of variance summarized by each dimension. The socio-demographic dimension of the vulnerability, accounting for the largest part of the variability, dominates the spatial pattern of the final vulnerability map. The Western part of the coast is generally characterized by low vulnerability, except for few unions surrounding the Sundarban mangrove, due to their poor access to basic facilities (Figure 4). Unlike other districts in the northern part of the study area, unions of Gopalganj district are characterized by exceptionally high vulnerability scores. This district is reported in the literature as a destination of people displaced by cyclone *Aila* [73] and seasonal agricultural laborers [74], as well as more limited access to safe drinking water [3]. High vulnerability might be due to these phenomena as well as a lower availability of high quality housing condition and a high proportion of disability documented in this study. Unions of the central coast that are adjacent to the sea or located along the major river have medium to very high vulnerability. Although the area around Chittagong city along the Southeast coast has generally a low vulnerability, the Kutubdia Island and a strip of mainland unions located opposite to the island are ranked as highly vulnerable. Unions of Teknaf upazilla, located in the land’s end at south of eastern coast, also received ranking of very high vulnerability. Very high vulnerable areas include major cities, such as Cox’s Bazar; some tourist attractions such as Maheshkhali and Teknaf; and some unions in islands such as Kutubdia and Hatiya.

### 4.3. Built Capacity against Cyclone

**Figure 5:**
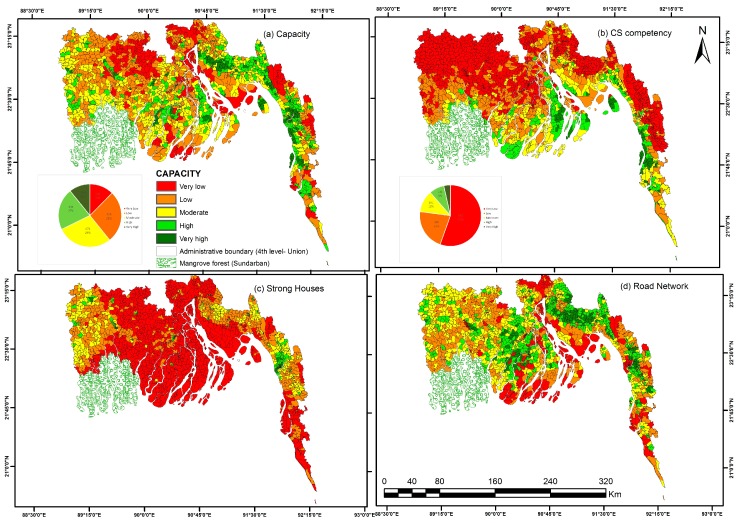
Capacity Index Map of the study area. (**a**) The Capacity index has been calculated by weighted (equal weight) sum of: (**b**) the cyclone shelter competency index; (**c**) the proportion of strong houses; and (**d**) the road network density. Cyclone shelter competency index of each union has been built using cyclone shelter related indicators.

The cyclone shelter competency map (Figure 5b) prepared from the cyclone shelters-related indicators shows that areas further away from the sea have very low shelter capacities (55% of the total area). These unions have no cyclone shelters or very scattered ones. This may be the result of limited casualties from historical cyclones in those places, leading to less attention of the government to build cyclone shelters in those areas. Not surprisingly, all the unions of Kutubdia Island have very high competency as it has received a lot of attention from researchers, media and donor agencies to build more cyclone shelters after the devastating cyclone in 1991. In general, most unions of the central and southern coast areas bordering the sea are well serviced by cyclone shelters, although this is not the case for all unions in the central coastal region. Lower values of the shelter competency index are obtained in the western coastal region, including for most unions directly bordering the Sundarban mangrove.

The capacity map (Figure 5a) considering cyclone shelter indicators, strong houses and the road network density shows a different spatial pattern than the cyclone shelter competency map. Indeed, strong houses are more common in the North West part of the coastal region as well as around Chittagong, but are very uncommon in the other part of the coastal area. In addition, the density of the road network is more evenly distributed across the coastal area, except for Unions located closest to the coast in the Central and Southern areas and along the Meghna River. Therefore, the overall built-up capacity against cyclone is not controlled by the distance to the coast. Unions in western parts of the coast are dominated by moderate to very high capacity except Gopalganj and Narail districts. Some parts of Bhola and Noakhali districts that have boundary with open sea are having very low capacity. Most unions in the southern coast area are characterized by high to very high capacity, including offshore Kutubdia Island. Very low capacity also exists in the northern part of central coast, in Sitakunda district and in the hilly parts of Chittagong district.

### 4.4. Physical Exposure to Cyclone and Tidal Surge

**Figure 6:**
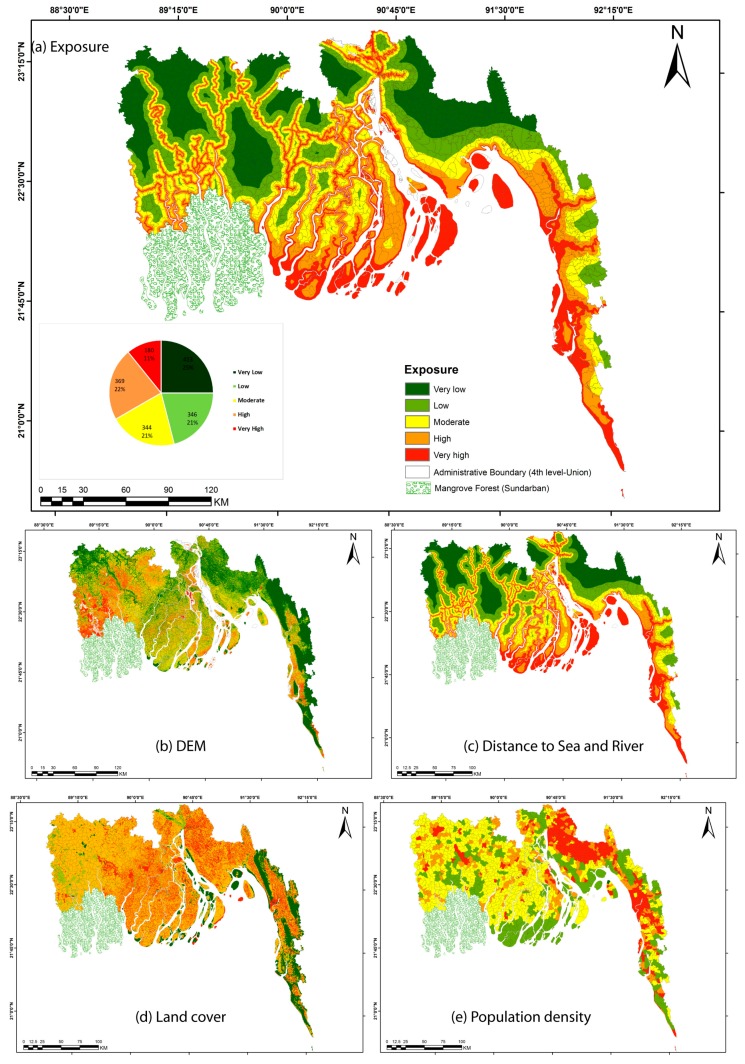
Exposure map of the study area. Exposure index has been calculated by equal weighted sum of four exposure indicators: DEM (**b**); distance to sea and river (**c**); land cover (**d**); and population density (**e**).

The pattern within the exposure map appears to be controlled mainly by the distance from sea water boundary (Figure 6a). All the offshore and riverine islands are highly exposed. The nearest places from water either be it rivers or open sea is more exposed than the places further away. Similar pattern of exposure in land cover and elevation map is visible in the eastern coast but they are not controlling the final exposure map (Figure 6b,c). Very high exposure to cyclone located mainland in western part is following the trails of inland river courses. The spatial pattern of population density and land cover is not strongly influencing the spatial pattern visible in Figure 6a but does control the local variation of values within each class.

### 4.5. Spatial Variation of Hazards

**Figure 7:**
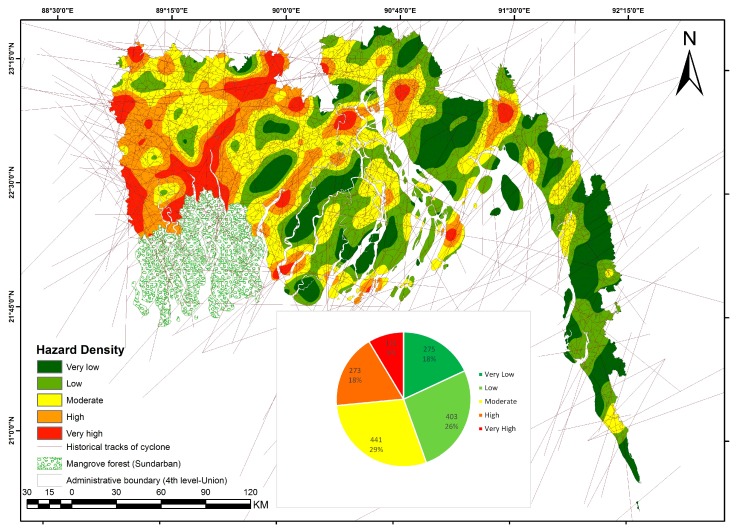
Hazard map of the study area. Cyclone hazard density was calculated by using cyclone tracks considering all cyclone categories that crossed Bangladesh in the period 1877–2015. Data have been compiled from NOAA IBTrACS (International Best Track Archive for Climate Stewardship), Global Risk Data Platform of UNISDR, Bangladesh meteorological department and published cyclone map of Banglapedia.

Hazard assessment is not easy in regions with scarce data such as Bangladesh. Characterizing hazard in the case of cyclone involves information on the frequency and track of cyclones, the wind speed at different locations (i.e., open sea, landfall site and inland), as well as the spatial variations of height of storm surge water. The only available parameter for historical cyclones of Bangladesh is the tracks, without information on the wind speed distribution, radius, etc. Figure 7 shows that cyclone track density is the highest in the western coast near Sundarban region. Islands and hilly areas of the southern coast are out of high and very high hazardous zone. Hatiya Island is also characterized by high to very high hazard.

### 4.6. Assessed Cyclone Risk

As we have low level of confidence on the hazard map, as it is produced based only on cyclone tracks, we formulated cyclone risk applying four kinds of mathematical combination of the vulnerability, exposure, hazard and capacity components. Standardized values of vulnerability, exposure, hazard and capacity are −1.13 to 1.13; −7.39 to 0.71; −2.17 to 3.39 and −1.1 to 2.13 respectively (Table 2). We used quantile as a cut point systematically to all of the produced maps to make them comparable and avoid the impact of aggregation method on the magnitude of risk and their components.

#### 4.6.1. Equal Hazard Risk: Exposure and Vulnerability

**Figure 8:**
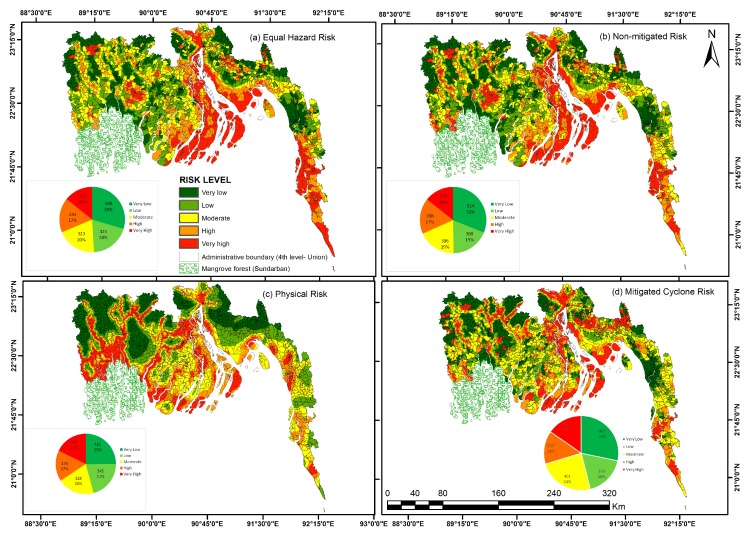
Risk Map of the study area: (**a**) Equal hazard Risk index was calculated by multiplying exposure and vulnerability; (**b**) Non-mitigated Risk index was calculated by multiplying hazard, exposure and vulnerability; (**c**) Physical Risk index was calculated by multiplying hazard and exposure; (**d**) Mitigated Risk index was calculated by multiplying hazard, exposure, and subtract of capacity from vulnerability.

This risk map takes into account the differential social indicators that cause certain populations to be more vulnerable to natural hazards as well as the physical factors that make the place differentially exposed to water surge and cyclone wind. Figure 8a highlights that unions within the delta of Meghna River are at very high risk. It includes almost all islands of central coast and the unions along both banks of Meghna River. Unions of Cox’s Bazar and Teknaf in the southern part of the east coast are also at very high risk as they associate a high physical exposure and a vulnerable population. Clusters of high risk unions are also noticed in the Western coast area, corresponding with urban centers gathering a high density of moderately vulnerable population.

#### 4.6.2. Non-Mitigated Risk: Hazard, Exposure, Vulnerability

Multiplying the map of “equal hazard risk” with the mapped distribution of cyclone hazard enable to generate the map of potential cyclone risk (Figure 8b). It shows a similar spatial pattern of risk as the map of general potential risk. The only difference is that potential cyclone risk is relatively higher in the western coast as the density of cyclone tracks is higher there. Very high risk zones are still observed along both banks of Meghna River but the amount of very high risk unions is higher in the northern part of the delta. Along the southern coast, the cyclone potential risk is generally lower than the rest of the coastline due to the lower density of cyclone tracks in that part of the study area.

#### 4.6.3. Physical Risk: Hazards and Exposure

Assuming equal socio-economic status of the coastal population, physical risk is the measurement of risk by taking into account the spatial distribution of hazard and exposure. The distribution of risk in the map of natural risk (Figure 8c) is displaying a different pattern compared to the two previous risk maps. Very high risk areas include unions along the sea coastline and along the banks of the main rivers in the central and western coastal zones, as well as in unions surrounding the Sundarban mangrove reserve. Chandpur, Noakhali and Feni districts, in the western part of central coast, are characterized by generally lower natural risk.

#### 4.6.4. Mitigated Cyclone Risk: Hazards, Exposure, Vulnerability and Capacity

Built capacity to face cyclones is partially balancing the vulnerability of the exposed population. The map of mitigated risk, incorporating capacity index with vulnerability, exposure and hazard index, shows a more complex spatial pattern than the non-mitigated risk. Most sea facing unions or islands in the central coast remain in the very high cyclone risk, together with a majority of riverine islands and unions along the banks of Meghna River. However, comparison of the maps shows that the high capacity of some unions in delta of Meghna River and in Cox’s Bazar district contribute in reducing their risk.

Table 2 shows the correlation among the four components that are used to calculate the risk maps to check their associations among themselves and with four risk maps. There is weak positive correlation between vulnerability and exposure and negative correlation of vulnerability with hazard and capacity. No correlation is found between built capacity and non-mitigated risk. However, there is a moderate positive correlation between the cyclone shelter competency index and the exposure; vulnerability and non-mitigated risk; and cyclone shelter competency and equal hazard risk. Among the risk indices, there is strong positive and significant correlations among equal hazard risk, non-mitigated risk and mitigated risk.

### 4.7. Validation of Risk Assessment

**Figure 9:**
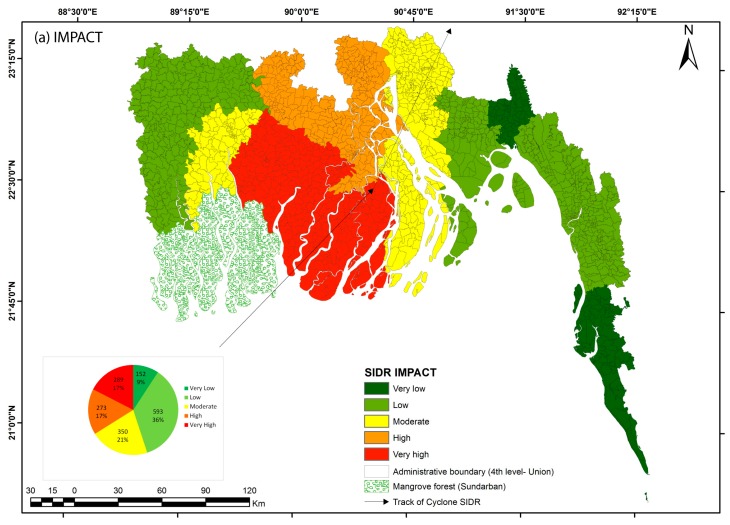

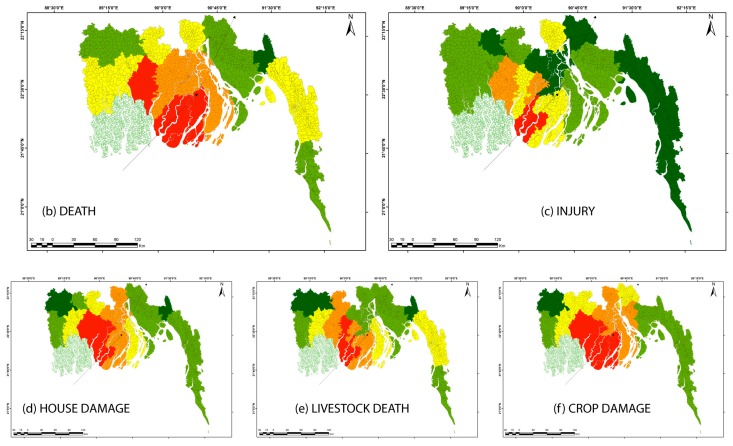
Map of impact of cyclone *Sidr*. Report of damage assessment of cyclone *Sidr* prepared by district commissioners were used to generate damage data and calculate damage index. Five components of loss or damage: (**b**) Death; (**c**) Injury; (**d**) house; (**e**) Livestock; and (**f**) crop have been ranked to the degree of damage and summed to produce the impact map (**a**).

The most challenging task in such regional risk assessment is the validation. The quality the risk assessment cannot be verified if the reliability of the produced risk map cannot be checked against damage data from historical disasters. Unfortunately, damage data are rare, causing most risk assessments proposed in literature not to be validated. We used a cyclone impact map based on cyclone *Sidr* as a proxy to compare our vulnerability and risk maps. Impact of cyclone *Sidr* is highly visible along its track in all component of direct loss (Figure 9). Very high impacted area are the contribution of highest number of human death and area of crop damage (Figure 9a,b,f). Visual comparison of vulnerability and *Sidr* impact map shows that low vulnerable unions of the western coast were less affected by cyclone *Sidr*, as the track of that cyclone was far away from that area (Figure 4 and Figure 9). Vulnerable unions (considering both in combined and demographic vulnerability) living in the central coast were moderately to very seriously impacted by cyclone *Sidr*. Pearson correlation shows a weak but significant positive correlation of vulnerability values to *Sidr* impact (Table 2). Comparison of risk and impact maps show that the places of very high death and injury by cyclone *Sidr* in south part of central coast matches with very high risk areas of equal hazard and non-mitigated risk maps. There is no other similar pattern in the impact maps and risk maps visible in other coastal places. Correlation matrix shows a weak positive but significant correlation between *Sidr* impact and physical risk map (Table 2). This can be accounted for by the fact that the impact of a single cyclone is strongly controlled by the spatial variation of cyclone intensity across and along its track, which is not accounted for in our risk maps. The damage data we used to map the impact of cyclone *Sidr* are based on the damage report prepared at the district level: this prevent us to compare quantitatively our vulnerability and risk maps that are produced at union scale. More importantly, the impact map being produced by a single cyclone, its spatial pattern is strongly controlled by the cyclone track as well as a decrease in intensity from the coastline to the inland. It is therefore impossible, based on the single-event impact data, to make any quantitative statement about the validity of our risk maps. We would need damage data at the same spatial administrative scale for a large number of historical cyclones to validate our maps. Alternative detailed damage data for a single cyclone across unions located close to each other—and therefore subjected to similar hazard intensity—but contrasted in their exposure and vulnerability characteristics could be useful to validate our regional approach.

## 5. Discussion

Risk assessment of natural hazards has been done previously in Bangladesh, but national scale assessments are generally restricted to province or district disaggregation level [75]. Most of the studies assessed vulnerability and risk at local level either in urban centers or in a localized small area [76,77]. Here, we analyzed data at the 4th administrative level for the whole coast of Bangladesh as it is the lowest administrative unit for which data exist. This is the relevant scale of analysis as it is lowest administrative level at which structural (i.e., cyclone shelters) and non-structural (i.e., unit of cyclone preparedness program and union disaster management committee) risk reduction measures are implemented. This is the first attempt to assess the lives and livelihood risk of Bangladesh coast to cyclone at union level. We considered all available variables that are considered as threat factors of lives (i.e., age, disability, housing condition, etc.) and livelihood (i.e., profession, unemployment, etc.) in the past disasters in Bangladesh. As this study used population census data of 2011, the assessed risk is a snapshot of risk at that year and should be updated with the latest available census data for assessing the risk of future cyclones.

### 5.1. Vulnerability of Coastal Bangladesh to Cyclone

Studies that assessed vulnerability of coast in different parts of the world used deductive methods (using limited indicators based on objective indicators available in literature) and inductive methods (aggregating many available indicators) to select the social indicators that influence hazard impacts [78,79]. We chose inductive method based on the available data and the lack of broadly accepted vulnerability indicators for Bangladesh. Although the factors extracted by PCA are not unique in all the social vulnerability calculations around the world, socio-economic characteristics such as gender, education, age, ethnicity, marital status, etc. are common in most of them. Those common variables are included in our first vulnerability dimension group. The role of variables such as the family size, the ratio of dependent people on the overall vulnerability index construction are justified by literature [10,56,68]. For all factors, the actual sign was not always obvious, as several parameters, including some with ambiguous relation with vulnerability, had significant loading.

High to very high vulnerable unions in central coast are characterized by the riverine and offshore island geographies. It is interesting to note that those islands are not only exposed to more severe natural processes, due to their low elevation and proximity to water, but they also concentrate vulnerable populations. Indeed, those islands have poor access to modern facilities, basic education, strong housing structures [48]. As an example, 75% of the population of Bhola Island was killed by the 1970 cyclone [80]. The South part of eastern coastal zone, including Cox’s Bazar district and Kutubdia Island, is dominated by high to very high vulnerability, for both its socio-demographic characteristics and its overall vulnerability. This results in relatively high potential risk values, despite a relatively lower hazard density. These are the areas which were severely affected by cyclone Gorky in 1991 [81,82,83]. The very high vulnerable unions surrounding the Sundarban reserve forest includes the few unions of Satkhira and Khulna districts. These two districts were highly affected by cyclone *Sidr* and *Aila* in 2007 and 2009, respectively [84,85].

The use of census data in vulnerability assessment involves some limitations. It includes the absence of meaningful vulnerability indicators such as income, language, knowledge on disaster etc. Using an inductive approach, the data reduction and aggregation technique is driven by the available data and its structure, more than a priori knowledge on factors controlling the vulnerability of local communities in the studied areas. Although the derived factors and their respective controlling variables are interpretable and consistent with the literature, it is difficult to know if the factors explaining the largest variance have indeed more relevance for the vulnerability index or if it is a mere consequence of the availability of redundant variables in the dataset. Direct validation of this vulnerability indicator is not possible at national scale, even if detailed disaster impact would be available, as impacts would be strongly influenced by spatial variation of hazard intensity and exposure as well.

### 5.2. Patterns of Built Capacity in Comparison with Vulnerability

The pattern of physical capacity, assessed in our method does not match the pattern of vulnerability in the coast of Bangladesh. There is even a weak negative correlation between the map of vulnerability and capacity (Table 2), meaning that highly vulnerable unions tend to have lower capacities. This is a direct consequence of our definition of the physical capacity as an aggregate of the cyclone shelter competency, the proportion of strong building and the road density. Indeed, there is a moderate positive correlation between the vulnerability index and the cyclone shelter competency index. Visual comparison of the two maps confirm that cyclone shelters in the coast of Bangladesh have effectively been constructed in the zone with the most vulnerable population, but also in the most exposed area (correlation 0.4). This confirms that cyclone shelters have been instrumental in reducing the number of fatalities of cyclones in these most risky areas [4,28,86]. Unions with high potential risk and highly vulnerable population are however in general having very poor housing structure and a limited density of road network (Figure 4a and Figure 7c,d), reducing the capacity of these Unions to face the adverse impact of cyclones. The integration of other potential capacity indicators therefore highlight that, if the development of infrastructures specifically targeting reduction of cyclone risk have effectively taken the distribution of cyclone risk into account, this is less the case of other physical infrastructures. Building qualities and road network are however deemed important elements for reducing the impact of cyclone disasters [77,87], although their importance relative to cyclone shelter is not still assessed. The positive correlation of our cyclone shelter competency index and exposure index indicates that the selection of location of cyclone shelters, at the scale of the entire coastal region, was based on exposure, as argued by CDMP (2009) and Mallick (2014) [49,57].

We are aware that the capacity to face cyclone disasters does not rely exclusively on physical infrastructures. The social and economic capitals of the exposed population are partially taken into account in the vulnerability assessment. The aspects related to the scientific forecasting and risk management, including evacuation order, are not considered in this study as no data enable to grasp these processes at the Union scale, but these are not expected to vary at a local spatial scale. Contrast in the socio-political context, i.e., local decision process, corruption, community support, that might affect the outcome of a disaster at local level can however not be grasped in our geo-statistical approach [88].

### 5.3. Exposure and Hazard Assessment

Although spatial contrast in cyclone impact on human lives and livelihoods can partly be attributed to differential socio-economic vulnerability of people living along the coast, the role of exposure and hazard, controlling the local intensity of the hazardous process (i.e., water depth, wind speed) is also important in controlling the impacts. Selection of variables, defining exposure and hazard, in this study is in agreement with Dwarakish et al. (2009) [60]. He used subjective ranking of six physical indicators to calculate coastal vulnerability index along the west coast of India. The aggregation method of the different exposure indicators was however modified in our study to account for a wider value range of the different indicators, for which a standardization and summation was deemed most appropriate, rather than a subjective ranking of individual indicators before aggregation. Exposure of individual union is mostly controlled by the distance to the sea and rivers as most of the coast is flat with the exception of the eastern coast around Chittagong (Figure 5b). Distance from sea is also taken as variable to assess exposure in Sousa et al. (2013) [58]. As for most historical cyclones in Bangladesh, tidal surge was the most important process causing fatalities, we integrated distance from river with that of sea considering the behavior of water surge through the rivers.

We used the density of cyclone tracks to roughly assess the spatial distribution of cyclone hazard across the coastal region. This approach is affected by significant limitation inherent to the limited available data. Buffer with arbitrary distance from historical cyclone tracks is used in hazard assessment in Esnard et al. (2011) [7]. All the cyclones, including low intensities ones, were taken into consideration, assuming that even low intensity cyclones can cause casualties through uplifted roofs of weak houses, uprooted branches of trees and drownings [86,89]. A cyclone loses its intensities as it travels over the land. Impact of storm surge is also reduced by forests and polders in the coastal area. Height of storm surge water associated with cyclone wind controls the potential damage by cyclones [64]. The non-availability of relevant data to document the spatial variability of these processes prevented to construct a hazard map considering these physical processes. However, consideration of the distance to sea and river as well as elevation in the exposure map enables grasping some of the local variations in the intensity of cyclone processes.

### 5.4. Mitigation of Cyclone Risk

Accurate risk assessment is important to support the development of effective cyclone mitigation policies and implementation of specific measures. The produced maps might contribute to improve three kinds of mitigation measures in Bangladesh, namely expand coverage of CPP (Cyclone Preparedness Programme) service areas, building new coastal embankments and construction of new cyclone shelters. The construction of embankments and cyclone shelters should be in the places with high and very high risk. Although successful early warning of some previous cyclones has been claimed to have saved many human lives, there is still controversy about false warning [83]. Incorporating the spatial distribution of vulnerable people in warning messages may increase trust of people to the warning signals. For example, the same danger level of cyclone warning is not expected to impact similarly places with strong or weak houses. The produced vulnerability and risk maps will give a basis to incorporate the characteristics of people in the future warning messages.

## 6. Conclusions

As Bangladesh is one of the most disaster prone countries, it is hit regularly by severe to very severe cyclones. Assessing the spatial distribution of risk is crucial to develop target risk management strategies and reduce impacts in term of casualties and property loss. This study aimed for the first time to identify the spatial variations and contributing components of risk for human lives and livelihood from cyclone and associated tidal surge. This integrated risk assessment incorporated a wide range of variables under the component of vulnerability, exposure, hazard and capacity. The method developed included exploratory inductive technique in vulnerability assessment, deductive technique in hazard, exposure and capacity assessment. Risk was assessed by simple equation considering the product of vulnerability minus the capacity, exposure and hazard.

Bangladesh being a developing country, the coastal inhabitants do not get the opportunity to subscribe life or property insurances. The government has no special allocation in its annual budget targeting the cyclone losses. To ensure an efficient use of the rehabilitation funds provided after each disaster by international donors, it is essential to assess the long term spatial distribution of casualty and livelihood risk of the coastal people to cyclones and not only invest all risk reduction actions in the areas that were most affected by the last cyclone. This research could therefore serve as a baseline document both at pre and post disaster phase for local and national disaster managers to adopt and efficiently implement structural and non-structural disaster risk reduction measures. The produced cyclone risk maps give a picture of the risk across the whole coast of Bangladesh based on the available data at the most detailed spatial scale. They enable highlighting the complex interplay of geographic, socio-demographic and physical infrastructure factors that contributes to the accumulation of risk in certain areas. The spatial pattern of vulnerability, capacity, exposure, hazard and risk may be the basis of the formulation of specific risk reduction strategies to face cyclone hazard. The method used in this study may be used with the best available similar types of data in risk assessment of other natural hazards in Bangladesh and other parts of the world. The use of four formulations of the risk in the same case study is useful to calculate different risk scenarios. It enables users to compare risk maps of different alternative scenarios, identify the major components controlling the risk gradients and take proper actions to improve the effectiveness of mitigation measures. Future works may include validation of risk maps using impact dataset of more historical cyclones. Development of the method should include more detailed studies on the factors controlling the spatial distribution of the experienced hazard intensity and the recorded direct and long term cyclone impacts on communities of the coastal regions of Bangladesh.

## Figures and Tables

**Table 1:** Socio-Economic variables and their directional effects on Vulnerability.

Components	Eigen Value	% Variance (Cumulative)	Weight	Directional Sign	Indicators	
Access to housing, resources, paid jobs, safe water and education (a)	7.59	19.5	0.25	−	Percent of male works in agriculture	−0.880
					Percent of male in service	0.873
					Percent of poor housing structure	−0.829
					Percent of tenants	0.817
					Percent of electricity connection	0.774
					Percent of female works in agriculture	−0.774
					Percent female in service	0.729
					Percent of “semi-pucka” housing structure	0.668
					Percent of people who drink water from pipeline supply	0.608
					Percent of employed female	0.589
					Percent of female engaged only in household work	−0.577
					Literacy rate	0.571
Household size, female marital status and dependency ratio (a)	3.52	28.5	0.11	+	Average size of the households	0.901
					Ratio of small family size over large ones	−0.852
					Ratio of unmarried female	0.848
					Dependency ratio	0.604
Gender specific employment and schooling status (a)	3.5	37.5	0.11	+	Percent of female who does not work	0.851
					Percent of male who does not work	0.831
					Percent of employed male who are at the age of school going but not attending school	−0.8
					Percent of Population who are not attending school even they are at the age of school going	−0.603
Physical, speech and overall disability (c)	2.07	42.8	0.07	+	Percent of total disable people	0.910
					Percent of physically disable people	0.851
					Percent of dumb people	0.593
Sex ratio and educational attainment of male (a)	1.92	47.7	0.06	+	Sex Ratio	0.875
					Percent of male who are not attending school even they are at the age of school going	0.702
Unsafe source of drinking water (b)	1.88	52.5	0.06	+	Percent of people using unconventional sources of drinking water	0.87
					Percent of people drinks water from tube-well	−0.836
Male and Female workers in Industry (a)	1.83	57.2	0.06	−	Percent of female works in industry	0.891
					Percent of male works in industry	0.785
Male and female job seekers (a)	1.75	61.7	0.06	+	Percent female looking for work	0.902
					Percent of male looking for work	0.863
Unhygienic toilet facilities (b)	1.57	65.7	0.05	+	Percent of people using non-sanitary toilets	−0.785
					Percent of people using non water-sealed sanitary toilets	0.760
Minority and ethnicity (c)	1.43	69.4	0.05	+	Percent of religious minority	0.719
					Percent of ethnic Population	0.655
Mental disorder (c)	1.29	72.7	0.04	+	Percent of people suffering from mental disorder	0.762
Autistic and hearing-impaired people (c)	1.24	75.9	0.04	+	Percent of Autistic people	0.798
					Percent of deaf people	0.616
Visually impaired people (c)	1.06	78.6	0.03	+	Percent of blind people	0.876

“−” denotes a decrease in vulnerability and “+” denotes an increase of vulnerability. Weight in % (Wi) = $\frac{variance}{total\;variance}\times 100$. Values after indicator denotes loading factor of each indicator. Letters in parentheses after each factor of first column represent three characteristics groups (Dimensions) of social vulnerability: (a) Demographic; (b) Basic facilities; and (c) Disability (See Figure 4).

**Table 2:** Correlation matrix among vulnerability, exposure, capacity, risk and *Sidr* impact maps. Pearson correlation has been calculated as pixel level. Numbers in parentheses refers to the ranges of values of the components.

Name of the Maps	Exposure (E, −7.39–0.71)	Hazard (H, −2.17–3.39)	Vulnerability (V, −1.13–1.13)	Capacity (C, −1.1–2.13)	CS Competency (CsC)	Equal Hazard Risk (Reh) E * V	Non-Mitigated Risk (Rn) E * H * V	Physical Risk (Rp) H * E	Mitigated Risk (Rm) E * H * (V–C)
H	−0.12 **								
V	0.16 **	−0.23 **							
C	−0.03	−0.02 **	−0.26 **						
CsC	0.40 **	−0.18 **	0.30 **	0.39 **					
Reh	0.40	−0.20 **	0.31 **	−0.08	0.33 **				
Rn	0.38	−0.17 **	0.26 **	−0.07	0.25 **	0.92 **			
Rp	0.93 **	−0.11 **	0.34 **	−0.01	0.34 **	0.37	0.43		
Rm	0.38 *	−0.12 *	0.21 **	0.21 **	0.15	0.68 **	0.75 **	0.43	
*Sidr* impact	0.12 **	0.02	0.10 **	−0.13 **	0.05 *	0.06 *	0.05 *	0.12 **	0.01

* Correlation is significant at 0.05 level (2-tailed), ** correlation is significant at 0.01 level (2-tailed).

## Supplementary Materials

The following are available online at www.mdpi.com/1660-4601/14/8/831/s1, Table S1: The description of the land covers classes and their relative ranking to exposure.
