# Supplementary material for: Assessing Risks from Cyclones for Human Lives and Livelihoods in the Coastal Region of Bangladesh
Source: Int J Environ Res Public Health. 2017 Jul 25;14(8):831. doi: 10.3390/ijerph14080831 (PMC5580535; doi:10.3390/ijerph14080831)
Supplement: Supplementary file 1 [file ijerph-14-00831-s001.pdf]

International Journal of
Environmental Research and Public Health

MDPI

Supplementary Materials

Assessing Risks from Cyclones for Human Lives and Livelihoods in the Coastal Region of Bangladesh

Mohammad Abdul Quader [1,2,*], Amanat Ullah Khan [3] and Matthieu Kervyn [1]

Table S1. The description of the land covers classes and their relative ranking to exposure.

**Land cover classes**	**Description**	**Relative exposure to cyclone and tidal surge***
Cultivable land	Land used for agriculture, dry firm land	7
Forest	Land cover with trees and vegetation cover over 30%	3
Grass land	Natural grass over 10%	5
Shrub land	Land covered with shrubs over 30%	4
Wet land	Land covered with wetland, trees, mangroves	2
Water bodies	Water bodies in the land area including river and pond…	6
Artificial surface	Land modified by human activities including settlement, industrial area, transport facilities, interior urban green zones and water bodies	8
Barren land	Land with less than 10% vegetation cover, saline and alkaline land, sandy field	1

*1 denotes lowest exposure
